# *Caenorhabditis Elegans*—Applications to Nematode Genomics

**DOI:** 10.1002/cfg.260

**Published:** 2003-04

**Authors:** William F. Gregory, John Parkinson

**Affiliations:** 1 Institute of Cell Animal and Population Biology Kings Buildings West Mains Rd Edinburgh EH9 3JT UK

## Abstract

The complete genome sequence of the free-living nematode *Caenorhabditis elegans* was published 4 years ago. Since then, we have seen great strides in technologies that seek to exploit this data. Here we describe the application of some of these techniques and other advances that are helping us to understand about not only the biology of this important model organism but also the entire phylum Nematoda.


**Comparative and Functional Genomics**
*Comp Funct Genom* 2003; **4**: 194–202.
Published online 1 April 2003 in Wiley InterScience (www.interscience.wiley.com). **DOI:** 10.1002/cfg.260

**Featured Organism**

### *Caenorhabditis elegans* — applications to nematode genomics

William F. Gregory* and John Parkinson
*Institute of Cell, Animal and Population Biology, Kings Buildings, West Mains Rd, Edinburgh EH9 3JT, UK*

**Correspondence to: William F. Gregory, Institute of Cell, Animal and Population Biology, Kings Buildings, West Mains Rd, Edinburgh EH9 3JT, UK. E-mail: b.gregory@ed.ac.uk*

Received: 28 January 2003
Accepted: 30 January 2003

### Abstract

**The complete genome sequence of the free-living nematode *Caenorhabditis elegans* was published 4 years ago. Since then, we have seen great strides in technologies that seek to exploit this data. Here we describe the application of some of these techniques and other advances that are helping us to understand about not only the biology of this important model organism but also the entire phylum Nematoda. Copyright © 2003 John Wiley & Sons, Ltd.**

**Keywords: nematode; genome; *Caenorhabditis elegans*; *Caenorhabditis briggsae*; RNAi; parasites; functional genomics**

### Background

*Caenorhabditis elegans* is a free-living soil nematode which has been developed as a model metazoan for over three decades (Brenner, 1974), work that recently resulted in the award of a Nobel Prize. *C. elegans* is a highly amenable experimental organism: it is relatively small (~1 mm in length), transparent, easy to culture and has a short generation time (~3 days). Furthermore, with the availability of its full genome sequence and with a host of well-developed experimental techniques *C. elegans* provides an ideal model for exploring the molecular aspects of a range of cell and developmental processes. Hence, as a model, it has proved invaluable in helping understand a number of biologically and medically important processes, including development, cancer, ageing and neurobiology (Boulton *et al.*, 2002; Coates and de Bono, 2002; de Bono *et al.*, 2002; Liu *et al.*, 2002; Partridge and Gems, 2002; Wodarz, 2002).

In addition to its role as a model metazoan, *C. elegans* is also the best characterized member of the phylum Nematoda. Phylogenetic analyses reveal that nematodes can be split into five major taxonomic groups (or clades) with *C. elegans* being a clade V organism (Blaxter, 1998; Dorris *et al.*, 1999). They are a highly diverse group of organisms occupying a diverse set of habitats, from obligate parasites of many forms of life, including plants, arthropods, fish and mammals to free-living organisms occupying niches such as soil and marine environments. It is interesting to note that parasitism appears to have evolved at multiple times during the evolution of the phylum (Dorris *et al.*, 1999).

### The *C. elegans* genome

Although over 97% of the 100 Mb *C. elegans* genome was available since the first publication in 1998, the full sequence was only finally finished at the end of 2002, making it the first truly complete animal genome. Analysis and annotation of the genome has been an ongoing process and will continue for the foreseeable future as new methods and data become available. Central to these analyses has been the WormBase consortium, a network of researchers involved in developing a web-based resource for the *C. elegans* genome (Harris *et al.*, 2003; Stein *et al.*, 2001). WormBase (available at **http://www.wormbase.org**, current release WS93b, 16 January 2003) is a continually evolving entity, incorporating not only the full genome sequence and associated gene models but also a

Copyright © 2003 John Wiley & Sons, Ltd.

whole wealth of other functional annotation (e.g. gene expression data, and double-stranded RNA knockdown experiments). WormBase is regularly updated and is being continually expanded as new data becomes available.

The *C. elegans* genome, split into five autosomes and one sex chromosome, is currently predicted to encode 21 197 proteins, of which 2927 are splice variants. Protein coding genes and RNA genes are initially predicted *ab initio* using the GeneFinder algorithm (Wilson *et al.*, unpublished; available at **http://ftp.genome.washington.edu/cgi-bin/Genefinder**). Genes are then further annotated in a manual process involving data from a range of different sources, including expressed sequence tag (EST)/mRNA sequence information, published papers and direct contact with the worm community. Interestingly, only ∼12 000 genes have been associated with ESTs despite the generation of over 191 524 ESTs (K. Bradnam, personal communication). This is probably due to the limited range of life cycle stages sampled by the cDNA libraries used to generate these sequences. Many genes may only be expressed at transient points in the life cycle or under specific conditions.

Protein-coding genes may be classified into three categories:

- Confirmed (3633 genes): there is transcript evidence for every base in every exon.
- Partially confirmed (8743 genes): there is some transcript evidence; however, the whole gene is not fully supported.
- Predicted (8821 genes): those without any transcript support.

Although gene prediction software is being continually developed, gene models associated with the last two categories may be inaccurate due to the lack of supporting data. The presence of pseudogenes (recently suggested to be as many as 20% of all annotated *C. elegans* genes; Mounsey *et al.*, 2002) further complicates the process of gene annotation. In a large-scale attempt to aid annotation, Reboul *et al.* (2001) are utilizing a systematic PCR-based approach to verify the gene model and expression of the open reading frames (ORFs) for each predicted gene. To date this approach has confirmed over 12 000 genes (Vaglio *et al.*, 2003) and current estimates argue for the existence of at least 17 300 genes. Furthermore, sequencing of the ORF-PCR products has helped correct the intron–exon structure for over a quarter of the predicted genes. There are also numerous non-protein-coding RNA genes: in addition to the easily recognizable rRNA, snRNA and tRNA genes, a number of small non-coding regulatory RNAs (termed microRNAs) have recently been discovered which appear to play an important role in regulating gene expression. Due to their small size (typically ∼22 bp), they are very difficult to recognize in the raw sequence and will therefore require sophisticated methods to identify such as the approach based on bioinformatics, comparative genomics and cDNA cloning used by Lee and Ambros (2001).

In terms of chromosomal organization, gene distribution between the chromosomes is non-uniform (see Table 1; *C. elegans* Sequencing Consortium, 1998). Genes appear to be denser towards the centre of the chromosomes, whilst tandem repeats and pseudogenes are more prevalent at the ends of chromosome arms. These findings suggest that these regions are evolving at a higher rate than the centre of the chromosomes (*C. elegans* Sequencing Consortium, 1998). In terms of gene organization, *C. elegans* and other nematodes appear unique among metazoans in having genes organized in operons (Blumenthal, 1998; Evans *et al.*, 1997; Guiliano *et al.*, 2002). These operons are resolved by *trans*-splicing: the first gene in the operon receives the spliced leader sequence (SL1), while downstream genes receive either SL1 or one of a family of alternative spliced leader sequences (SL2s). Whereas SL1 is added to many transcripts that are not part of operons, it seems that SL2s are only found on downstream genes in operons. A recent analysis using the occurrence of SL2 to define operons suggests that there are ∼1000 operons containing two to eight genes (Blumenthal *et al.*, 2002).

### The *C. briggsae* genome

This year saw the release of the first draft of the *Caenorhabditis briggsae* genome. *C. briggsae* is another free-living soil nematode thought to have diverged from *C. elegans* ∼50–150 million years ago (Coghlan and Wolfe, 2002). The current *C. briggsae* genome assembly is 102 MB, comprising 142 pieces, and is essentially 98% complete (**http://www.sanger.ac.uk/Projects/C_briggsae/**).

Copyright © 2003 John Wiley & Sons, Ltd.

**Table 1.** Distribution of the 21 197 predicted genes in *C. elegans* across the six chromosomes indicating their relative gene density, the proportion of genes giving an RNAi phenotype, and their homology to two different sequence databases

| Chromosome | No. of predicted genes | Length (Mb) | Gene density (genes/Mb) | RNAi phenotypes (% of genes) | Proteins with similarity to SWISS-TrEMBL[1a] (% of genes) | Proteins with similarity to *C. briggsae*[1b] (% of genes) |
|---|---|---|---|---|---|---|
| I | 3068 | 15.08 | 203.4 | 13.7 | 1949 (64) | 2788 (91) |
| II | 3692 | 15.26 | 241.9 | 11.9 | 1955 (53) | 3237 (88) |
| III | 2957 | 13.78 | 214.6 | 18.5 | 1853 (63) | 2665 (90) |
| IV | 3409 | 17.49 | 194.9 | 10.9 | 1958 (57) | 3085 (90) |
| V | 5082 | 20.92 | 242.9 | 5.2 | 2252 (44) | 4603 (91) |
| X | 2989 | 17.72 | 168.7 | 5.6 | 1635 (55) | 2567 (86) |

[1] Predicted proteins from the *C. elegans* genome were searched for sequence similarity using BLAST against two different databases: (a) BLASTP vs. SWISS-TrEMBL — a comprehensive protein sequence database which combines SWISS-PROT with the translation of all protein-coding sequences from the EMBL nucleotide sequence database. Nematode proteins were removed from the database before analysis; (b) TBLASTN vs. the *C. briggsae* draft genome. The numbers given are those proteins which had a raw BLAST score of >50 against the respective databases.

Initial analysis suggests that the coding regions are more similar to the *C. elegans* genome than the non-coding regions. Thus, in addition to *C. elegans* providing a springboard for annotation of the *C. briggsae* genome, identification of homologous sequences within their respective genomes offers a new method of gene identification for *C. elegans* (Lee and Ambros, 2001). Furthermore, Webb *et al.* (2002) have found that those intergenic regions that also share homology between the two genomes may also be involved in gene regulatory functions. BLAST analysis of the *C. elegans* protein dataset suggests that ∼10% of *C. elegans* genes do not share significant similarity with a *C. briggsae* gene and that these genes are spread equally between the six *C. elegans* chromosomes (our unpublished data; see Table 1). However, a lower proportion of predicted proteins from chromosome V appear to have a significant match to a sequence in the SWISS-PROT/TrEMBL database than those from other chromosomes. This may indicate that chromosome V contains a higher proportion of nematode-specific genes.

Global comparisons of sections of the *C. elegans* and *C. briggsae* genomes suggest that *Caenorhabditis* spp. have a rearrangement rate of 0.4–1.0 breakages/Mb/million years (Coghlan and Wolfe, 2002), which is at least four times faster than the rate in *Drosophila melanogaster*. These comparisons are an ongoing exercise and are expected to reveal many interesting insights into their evolution.

### Other nematode genomes

Many species of nematode pose a significant risk to human health and agriculture worldwide. This has led to the initiation of a number of sequencing projects aimed at elucidating some of the molecular aspects of parasitism. At present 33 different nematode species are the subject of EST sequencing projects (Parkinson *et al.*, 2001). In an attempt to place this sequence data in a genomic context, ESTs from each species are clustered on the basis of sequence similarity to form a non-redundant set of putative gene transcripts. Initial comparisons of the 'partial genomes' from a few species reveals that the degree of similarity between parasitic nematodes and *C. elegans* reflects their underlying phylogeny (unpublished data). For example, of the predicted genes from the mouse whipworm *Trichuris muris* (a clade I nematode) only ∼40% have significant similarity to a *C. elegans* gene, whilst of the predicted genes from the sheep hookworm *Haemonchus contortus* (a clade V nematode) ∼65% appear to share significant similarity with *C. elegans*. Although these figures for similarity may be artificially low due to the small number of cDNA libraries sampled, they do suggest that whilst *C. elegans* may serve as a useful model of clade V nematodes, transfer of genomic knowledge to nematodes from less related clades may prove problematic. In addition to these EST initiatives, last year the Institute for Genome Research (TIGR) received funding for a three-fold shotgun coverage of the

Copyright © 2003 John Wiley & Sons, Ltd.

110 Mb genome of the filarial parasite *Brugia malayi* (**http://www.tigr.org/tdb/e2k1/bma1**).

To date there is little information on the amount of synteny between *C. elegans* and related parasites. However, last year one study published a comparison of a genomic fragment from *B. malayi* with the syntenic region from *C. elegans*. Synteny was observed, with the order, composition and configuration of genes being conserved for putative orthologous genes (Guiliano *et al.*, 2002).

### Functional genomics

Less than 5% of the 21 197 predicted proteins encoded by the *C. elegans* genome have so far been characterized by conventional genetics or biochemisty. To redress this deficit of biological information over sequence information, several laboratories have initiated high-throughput functional genomic approaches based on generation of loss-of-function phenotypes, analysis of expression profiles and protein–protein interactions.

### RNA interference

In the 6 years since it was first discovered in *C. elegans* (Fire *et al.*, 1998), double-stranded RNA-mediated interference (RNAi) has become an integral part of many *C. elegans* laboratories and is often used as a first approach when investigating gene function. When compared with classical mutagenesis screens, inactivation of specific gene products provides a more rapid link between sequence data and biological function. Knockdown of gene expression can be provoked by injection of the dsRNA of interest into the gonad (Fire *et al.*, 1998), by soaking in dsRNA (Tabara *et al.*, 1998) or by feeding on dsRNA-expressing bacteria (Timmons *et al.*, 2001). Regardless of the route of administration, the RNAi effect is transported across cellular boundaries, so that its effect is seen throughout most of the worm and is transmitted to the offspring of treated worms. These effects are due to distinct mechanisms (Grishok *et al.*, 2000; Ketting *et al.*, 1999). Bacterial feeding, in particular, is an easy and efficient technique capable of being performed in most laboratories (Kamath *et al.*, 2001).

The knockdown of 16 757 genes (around 80% of the predicted gene complement) by RNAi has recently been carried out, representing the first near-complete targeting of a metazoan genome (Kamath *et al.*, 2003). RNAi phenotypes were assigned to 1528 genes, more than 1000 of which had not previously been annotated with a biological function. Overall, 63% of loci already known by mutation were detected by RNAi and gave similar phenotypes. Significant differences were detected in the types of genes resulting in 'non-viable' phenotypes (lethal and sterile phenotypes) and 'viable phenotypes' (post-embryonic phenotypes). *C. elegans* genes having clear orthologues in other eukaryotes were far more likely to produce a non-viable RNAi phenotype than non-orthologous genes, probably due to the fact that conserved genes tend to be essential. Viable phenotypes, on the other hand, were more likely to be related to genes of unknown function, underlining our current lack of understanding of metazoan development. Table 1 shows the distribution of phenotypes obtained across the six chromosomes. Significantly, genes targeted on chromosomes V and X are less likely to show a phenotype than those on the other chromosomes. In the case of chromosome V this may be associated with the greater number of genes that are nematode-specific (and hence less likely to perform essential functions). For chromosome X, many more genes are associated with worm behaviour or morphology and are thus less likely to show non-viable phenotypes.

A key advantage of this impressive study is the construction of a re-useable library of dsRNA-expressing bacteria, which is made available to other researchers via the HGMP Resource Centre (**http://www.hgmp.ac.uk**). The library has already been used to screen for genes involved in ageing (Lee *et al.*, 2003) and fat regulation (Ashrafi *et al.*, 2003).

In addition to RNAi, the *C. elegans* Gene Knockout Consortium has generated several hundred mutant lines using a PCR-based method to detect deletions in genes specifically requested from the *C. elegans* community (**http://elegans.bcgsc.bc.ca/knockout.shtml**). Practically, these mutants offer the advantage of generating stable mutant lines that can be maintained indefinitely.

Through these gene inactivation and deletion studies, we now know that the vast majority

Copyright © 2003 John Wiley & Sons, Ltd.

of genes yield no obvious phenotype. A further observation in RNAi screens is that only about two-thirds of genes already identified by classical mutational genetics give RNAi phenotypes. Although RNAi and deletion mutants are undoubtedly powerful techniques, they can only generate loss-of-function phenotypes. Classical forward genetics, on the other hand, has the ability to generate different mutant phenotypes depending on how it alters genetic loci, e.g. single nucleotide changes can result in more subtle phenotypes than deletions, and gain-of-function alleles can reveal the function of genes with redundant function.

### Expression profiling

Gene expression profiling using microarray technology is now a standard method for gaining functional information for unknown genes and to help understand aspects of gene regulation. One of the most widely used microarray tools for *C. elegans* is the resource provided by Stuart Kim's lab in UC Stanford, USA. RNA samples may be sent for analysis against microarrays produced in-house and the results are stored on their local database (**http://genome-www5.stanford.edu/MicroArray/SMD/**). Initial studies were performed on a microarray comprising 11 990 genes (155 experiments). More recently, a 'whole genome' microarray has been produced containing ~1 kb fragments from 17 871 genes (Jiang *et al.*, 2001). To date, more than 1000 experiments involving many growth conditions, developmental stages and varieties of mutants have been performed. The availability of a central database resource enables global analyses of all the experiments, revealing an expression landscape in which there are mountains of genes with related expression profiles (Kim *et al.*, 2001).

An alternative commercial *C. elegans* microarray is available from Affymetrix (**http://www.affymetrix.com/products/index.affx**). Many other companies also provide the option to construct custom arrays from either PCR products or synthesized oligonucleotides. Given the almost weekly changes in annotation to *C. elegans* genes and the low relative cost of manufacturing new microarrays, projects wishing to use large numbers of microarrays may wish to consider this latter option.

Recently, the filarial genome project has begun to construct a microarray containing 4000 *B. malayi* transcripts selected from EST datasets (Steve Williams, Smith College, MA, personal communication). Microarrays are expected to be available from March this year and will provide an exciting source of new data on gene expression in this medically important organism.

Aside from microarray technology, two groups are analysing gene expression with reporter constructs and *in situ* hydridization. Ian Hope's group has used a promoter trapping approach to assess gene expression (Hope *et al.*, 1998). Profiles of 342 genes are available on their website (**http://bgypc086.leeds.ac.uk/**). Yuji Kohara and colleagues are hybridizing a non-redundant set of cDNA clones from their EST sequencing project. Images are available on their website (**http://nematode.lab.nig.ac.jp/db/**).

### Interaction screens

A genome-wide yeast two-hybrid analysis aims to eventually provide a complete catalogue of protein–protein interactions (**http://vidal.dfci.harvard.edu/interactome.htm**). Initially the feasibility of such a high-throughput technique was tested by fishing for interactions within a set of 27 genes involved in vulval development (Walhout *et al.*, 2000). Eleven interactions between these genes had already been reported in the literature. By pair-wise matching each protein, six of these 11 interactions were detected. The failure to detect the remaining interactions is probably due to the inherent limitation of the two-hybrid assay (weak interactions may not be detected) or the physiology of the yeast cell (important post-translational modifications may not be performed correctly). Further interactions were sought by using the 27 proteins to screen a worm cDNA library, identifying a total of 148 interaction partners, of which only 15 had previously been described. Further work successfully identified the full complement of interacting partners in the proteasome (Davy *et al.*, 2001). The large amount of genome-wide expression, RNAi and interaction data is now beginning to be correlated (Walhout *et al.*, 2002). Finding correlates between these datasets helps validate the interaction data, e.g., in the yeast *Saccharomyces cerevisiae* potential interaction partners are more likely

Copyright © 2003 John Wiley & Sons, Ltd. 

to be co-expressed, and their knockdown phenotypes are often the same (Ge *et al.*, 2001). Conversely, clustering of genes by their phenotype (Piano *et al.*, 2002) or expression data (Roy *et al.*, 2002) can be used to hypothesize on the function of novel proteins within clusters where many genes have already been ascribed biological functions. These studies have also shown that clustered genes are found within groups along chromosomes (Roy *et al.*, 2002).

### Transgenesis in parasites

Much has been learned about basic nematode biology, and of some drug targets that have close counterparts in *C. elegans*. To understand the function of the many parasite-specific gene products, techniques developed by the *C. elegans* community are starting to be adapted in parasite studies (Hashmi *et al.*, 2001).

Several attempts at transgenesis in parasites have met with variable success (Davis *et al.*, 1999; Hashmi *et al.*, 1995; Jackstadt *et al.*, 1999; Lok and Massey, 2002). Microinjection and ballistic DNA transfer into several species have demonstrated promoter activity and protein production in injected parasites, but rarely in their progeny. Often transgene activity can only be seen around the site where the DNA was introduced, e.g. in *Strongyloides stercoralis*, a parasite of the human gut, injection of an actin–green fluorescent protein (GFP) construct gave rise to fluorescence only in gonadal tissues and released embryos (Lok and Massey, 2002). All of the GFP-positive embryos failed to hatch. Although many nematodes will prove refractory to microinjection techniques as developed for *C. elegans*, these techniques will continue to be developed and could provide valuable tools for parasite research in the future.

### Parasite genes in *C. elegans*

Promoter regions of parasitic genes have been shown to be active in *C. elegans*, but correct timing or location is not always seen (Britton *et al.*, 1999; Gomez-Escobar *et al.*, 2002). Orthology between *Haemonchus contortus* and *C. elegans* cysteine protease genes was suggested in studies that showed that the *Haemonchus* gene, under control of the *C. elegans* promoter, could functionally rescue a *C. elegans* mutant line (Britton and Murray, 2002). Expression of *Onchocerca volvulus* glutathione S-transferase (GST) in *C. elegans* demonstrated correct processing of the mRNA as well as signal peptide cleavage and glycosylation of the mature protein (Krause *et al.*, 2001), suggesting that this may be a useful system for producing parasite proteins.

### RNAi in parasites

Although tremendously successful in *C. elegans*, the use of RNAi is only just starting to be addressed in parasites. The short period of protein knockdown may well limit its use in parasites, especially those with extended developmental cycles. To date, RNAi by soaking has proved successful in the gut nematode *Nippostrongylus brasiliensis* (Hussein *et al.*, 2002), the filarial nematode *B. malayi* (Aboobaker and Blaxter, in preparation) and two plant parasitic nematodes *Heterodera glycines* and *Globodera pallida* (Urwin *et al.*, 2002). Suppression in *N. brasiliensis* lasted for at least 6 days after exposure to dsRNA for 16 h. This length of knockdown is sufficient to assess the survival of treated worms once they are reintroduced into their animal host. A similar method was used to suppress a gene required to form the protective sheath around the first larval stage of *B. malayi*; this resulted in a drastic reduction in the number of released larvae (Aboobaker and Blaxter, in preparation).

### Conclusions

This review summarizes the techniques currently used to assess gene function in *C. elegans* and other species of nematodes. Many of the techniques that have been developed are high-throughput, but can easily be adapted to analyse single genes, or groups of genes, in more detail. The data and reagents already available represent an advanced starting point for many projects. The *C. elegans* model is also a foundation for studying protein function in many medically and economically important nematode species, and has already helped define what genes may encode species-specific proteins in other nematodes. In the future, molecular and

Copyright © 2003 John Wiley & Sons, Ltd. 

structural studies will help define the function of many nematode proteins and their interactions within large biological networks.

### Web-based resources

### WormBase

**http://www.wormbase.org/**
The most comprehensive and up-to-date resource, covering many aspects of the *C. elegans* genome and its biology. The genome browser boasts a highly configurable interface, allowing the display of many features and annotations, including gene models, alignments to ESTs and the *C. briggsae* draft genome (and other sequences). In addition, WormBase includes a number of genome-wide data sets, including microarray expression data from a variety of life stages and conditions, RNAi experiments (16 757 genes), GFP expression data (covering 748 genes), single nucleotide polymorphisms (6386 in total) and cell lineage information. WormBase is an ongoing project; new releases are published every ∼2 weeks and will continue to develop and include new features as data becomes available.

### The Wellcome Trust Sanger Institute and the Washington University Genome Sequencing Center *C. elegans* pages

**http://www.sanger.ac.uk/Projects/C_elegans/**
**http://genome.wustl.edu/projects/celegans/**
These two centres were jointly responsible for generating the majority of the *C. elegans* genome sequence. The pages provide information on the *C. elegans* sequencing project, access to the sequence data and links to a number of related sites. They also both host a BLAST server allowing the user to search available *C. elegans* sequence data (**http://www.sanger.ac.uk/Projects/C_elegans/blast_server.shtml** and **http://genome.wustl.edu/blast/client.pl**).

### The Wellcome Trust Sanger Institute and the Washington University Genome Sequencing Center *C. briggsae* pages

**http://www.sanger.ac.uk/Projects/C_elegans/**
**http://genome.wustl.edu/projects/cbriggsae/**
These pages provide information about the ongoing *C. briggsae* genome project, provide links to the sequence data, and also feature BLAST servers enabling the user to search the current *C. briggsae* genome data (**http://www.sanger.ac.uk/Projects/C_briggsae/blast_server.shtml** and **http://genome.wustl.edu/blast/briggsae_client.cgi**).

### The Institute for Genome Research (TIGR)

**http://www.tigr.org/tdb/e2k1/bma1/**
TIGR are currently funded to perform a three-fold shotgun coverage of the *B. malayi* genome. These pages provide information on the project, access to the sequence data (via public and licensed ftp sites), and also feature a BLAST server **http://tigrblast.tigr.org/er-blast/index.cgi?project=bma1**, which enables the user to search against the preliminary sequence assembly. In addition, TIGR have also generated sets of gene indices both for *B. malayi* and *O. volvulus* from the available EST data (**http://www.tigr.org/tdb/tgi/**)

### NEMBASE and NemaGene

**http://www.nematodes.org/nematodeESTs/nembase.html**
**http://www.nematode.net/NemaGene/index.php**
These related databases are currently being developed as part of the parasitic nematode EST initiatives. For a number of species of nematodes, sequences are clustered (on the basis of sequence identity) into groups that putatively derive from the same gene, to form 'partial genomes'. Web-based forms allow the identification of genes of interest via simple BLAST annotation or sequence similarity. Other features include the ability to define groups of genes on the basis of expression or similarity profiles (Parkinson and Blaxter, 2003).

### WorfDB

**http://worfdb.dfci.harvard.edu/**
A database created to organize the data associated with the cloning of the complete set of predicted protein-encoding open reading frames (ORFs). It allows remote users to search for the availability and quality of cloned ORFs and displays the ORFs in context with the gene predictions.

### WormPD

**http://www.incyte.com/sequence/proteome/databases/WormPD.shtml**

Copyright © 2003 John Wiley & Sons, Ltd. 

A commercially produced venture which attempts to collate all currently published information on *C. elegans*, along with a number of automated analyses, (e.g. BLAST searches against a variety of protein databases) into one central resource.

### NEXTDB: The Nematode Expression Pattern DataBase

**http://nematode.lab.nig.ac.jp/**
The Kohara lab has been constructing an expression pattern map of the *C. elegans* genome through EST analysis and systematic whole mount *in situ* hybridization. NEXTDB attempts to integrate all information from their expression pattern project.

### The Interactome Project

**http://vidal.dfci.harvard.edu/interactome.htm**
These pages describe the initial findings from Dr Marc Vidal's interactome project.

### *C. elegans* transposon insertion project

**http://www.cgmc.univ-lyon1.fr/**
Dr Laurent Segalat's lab is producing a transposon-based strategy to identify *C. elegans* mutants. These pages allow the user to search for a gene of interest and request any available mutants.

### *C. elegans* gene knockout project

**http://elegans.bcgsc.bc.ca/knockout.shtml**
This is a worldwide consortium whose goal is to produce null alleles of all known genes in the *C. elegans* genome. You can submit requests of genes to target, and browse the lists of genes for which an allele has already been generated (currently ~600).

### The Worm DNA Microarray Center

**http://cmgm.stanford.edu/~kimlab/wmdirectorybig.html**
The page provides information on Dr Stuart Kim's large-scale microarray-based gene expression analysis project for *C. elegans*. It also contains information on how to take part and how to access the data (stored at the Stanford Microarray database).

### *Caenorhabditis* Genetics Center

**http://biosci.umn.edu/CGC/CGChomepage.htm**
The center (situated in Minnesota) is responsible for collecting, maintaining and distributing stocks of *C. elegans*, maintaining a *C. elegans* bibliography, and publishing and distributing the *Worm Breeder's Gazette*. A team in Oxford is responsible for coordinating genetic nomenclature and maintaining the *C. elegans* genetic map. A team in Dallas is responsible for maintaining the *C. elegans* web server (**http://elegans.swmed.edu/**).

### References

Ashrafi K, Chang FY, Watts JL, *et al.* 2003. Genome-wide RNAi analysis of *Caenorhabditis elegans* fat regulatory genes. *Nature* **421**: 268–272.

Blaxter M. 1998. *Caenorhabditis elegans* is a nematode. *Science* **282**: 2041–2046.

Blumenthal T. 1998. Gene clusters and polycistronic transcription in eukaryotes. *Bioessays* **20**: 480–487.

Blumenthal T, Evans D, Link CD, *et al.* 2002. A global analysis of *Caenorhabditis elegans* operons. *Nature* **417**: 851–854.

Boulton SJ, Gartner A, Reboul J, *et al.* 2002. Combined functional genomic maps of the *C. elegans* DNA damage response. *Science* **295**: 127–131.

Brenner S. 1974. The genetics of *Caenorhabditis elegans*. *Genetics* **77**: 71–94.

Britton C, Murray L. 2002. A cathepsin L protease essential for *Caenorhabditis elegans* embryogenesis is functionally conserved in parasitic nematodes. *Mol Biochem Parasitol* **122**: 21–33.

Britton C, Redmond DL, Knox DP, McKerrow JH, Barry JD. 1999. Identification of promoter elements of parasite nematode genes in transgenic *Caenorhabditis elegans*. *Mol Biochem Parasitol* **103**: 171–181.

Coates JC, de Bono M. 2002. Antagonistic pathways in neurons exposed to body fluid regulate social feeding in *Caenorhabditis elegans*. *Nature* **419**: 925–929.

Coghlan A, Wolfe KH. 2002. Four-fold faster rate of genome rearrangement in nematodes than in *Drosophila*. *Genome Res* **12**: 857–867.

Davis RE, Parra A, LoVerde PT, *et al.* 1999. Transient expression of DNA and RNA in parasitic helminths by using particle bombardment. *Proc Natl Acad Sci USA* **96**: 8687–8692.

Davy A, Bello P, Thierry-Mieg N, *et al.* 2001. A protein–protein interaction map of the *Caenorhabditis elegans* 26S proteasome. *EMBO Rep* **2**: 821–828.

de Bono M, Tobin DM, Davis MW, Avery L, Bargmann CI. 2002. Social feeding in *Caenorhabditis elegans* is induced by neurons that detect aversive stimuli. *Nature* **419**: 899–903.

Dorris M, De Ley P, Blaxter ML. 1999. Molecular analysis of nematode diversity and the evolution of parasitism. *Parasitol Today* **15**: 188–193.

Evans D, Zorio D, MacMorris M, *et al.* 1997. Operons and SL2 trans-splicing exist in nematodes outside the genus *Caenorhabditis*. *Proc Natl Acad Sci USA* **94**: 9751–9756.

Fire A, Xu S, Montgomery MK, *et al.* 1998. Potent and specific genetic interference by double-stranded RNA in *Caenorhabditis elegans*. *Nature* **391**: 806–811.

Copyright © 2003 John Wiley & Sons, Ltd. 

Ge H, Liu Z, Church GM, Vidal M. 2001. Correlation between transcriptome and interactome mapping data from *Saccharomyces cerevisiae*. *Nature Genet* **29**: 482–486.

Gomez-Escobar N, Gregory WF, Britton C, *et al.* 2002. Abundant larval transcript-1 and -2 genes from *Brugia malayi*: diversity of genomic environments but conservation of 5′ promoter sequences functional in *Caenorhabditis elegans*. *Mol Biochem Parasitol* **125**: 59–71.

Grishok A, Tabara H, Mello CC. 2000. Genetic requirements for inheritance of RNAi in *C. elegans*. *Science* **287**: 2494–2497.

Guiliano DB, Hall N, Jones SJ, *et al.* 2002. Conservation of long-range synteny and microsynteny between the genomes of two distantly related nematodes. *Genome Biol* **3**: 0057.1–0057.14.

Harris TW, Lee R, Schwarz E, *et al.* 2003. WormBase: a cross-species database for comparative genomics. *Nucleic Acids Res* **31**: 133–137.

Hashmi S, Hashmi G, Gaugler R. 1995. Genetic transformation of an entomopathogenic nematode by microinjection. *J Invertebr Pathol* **66**: 293–296.

Hashmi S, Tawe W, Lustigman S. 2001. *Caenorhabditis elegans* and the study of gene function in parasites. *Trends Parasitol* **17**: 387–393.

Hope IA, Arnold JM, McCarroll D, *et al.* 1998. Promoter trapping identifies real genes in *C. elegans*. *Mol Gen Genet* **260**: 300–308.

Hussein AS, Kichenin K, Selkirk ME. 2002. Suppression of secreted acetylcholinesterase expression in *Nippostrongylus brasiliensis* by RNA interference. *Mol Biochem Parasitol* **122**: 91–94.

Jackstadt P, Wilm TP, Zahner H, Hobom G. 1999. Transformation of nematodes via ballistic DNA transfer. *Mol Biochem Parasitol* **103**: 261–266.

Jiang M, Ryu J, Kiraly M, Duke K, Reinke V, Kim SK. 2001. Genome-wide analysis of developmental and sex-regulated gene expression profiles in *Caenorhabditis elegans*. *Proc Natl Acad Sci USA* **98**: 218–223.

Kamath RS, Fraser AG, Dong Y, *et al.* 2003. Systematic functional analysis of the *Caenorhabditis elegans* genome using RNAi. *Nature* **421**: 231–237.

Kamath RS, Martinez-Campos M, Zipperlen P, Fraser AG, Ahringer J. 2001. Effectiveness of specific RNA-mediated interference through ingested double-stranded RNA in *Caenorhabditis elegans*. *Genome Biol* **2**: 0002.1–0002.10.

Ketting RF, Haverkamp TH, van Luenen HG, Plasterk RH. 1999. Mut-7 of *C. elegans*, required for transposon silencing and RNA interference, is a homolog of Werner syndrome helicase and RNaseD. *Cell* **99**: 133–141.

Krause S, Sommer A, Fischer P, *et al.* 2001. Gene structure of the extracellular glutathione S-transferase from *Onchocerca volvulus* and its overexpression and promoter analysis in transgenic *Caenorhabditis elegans*. *Mol Biochem Parasitol* **117**: 145–154.

Lee RC, Ambros V. 2001. An extensive class of small RNAs in *Caenorhabditis elegans*. *Science* **294**: 862–864.

Lee SS, Lee RY, Fraser AG, *et al.* 2003. A systematic RNAi screen identifies a critical role for mitochondria in *C. elegans* longevity. *Nature Genet* **33**: 40–48.

Liu Y, Dodds P, Emilion G, *et al.* 2002. The human homologue of unc-93 maps to chromosome 6q27 — characterisation and analysis in sporadic epithelial ovarian cancer. *BMC Genet* **3**: 20.

Lok JB, Massey HC Jr. 2002. Transgene expression in *Strongyloides stercoralis* following gonadal microinjection of DNA constructs. *Mol Biochem Parasitol* **119**: 279–284.

Mounsey A, Bauer P, Hope IA. 2002. Evidence suggesting that a fifth of annotated *Caenorhabditis elegans* genes may be pseudogenes. *Genome Res* **12**: 770–775.

Parkinson J, Blaxter M. 2003. SimiTri — visualising similarity relationships for groups of sequences. *Bioinformatics* **19**: 390–395.

Parkinson J, Whitton C, Guiliano D, Daub J, Blaxter M. 2001. 200 000 nematode expressed sequence tags on the Net. *Trends Parasitol* **17**: 394–396.

Partridge L, Gems D. 2002. Mechanisms of ageing: public or private? *Nature Rev Genet* **3**: 165–175.

Piano F, Schetter AJ, Morton DG, *et al.* 2002. Gene clustering based on RNAi phenotypes of ovary-enriched genes in *C. elegans*. *Curr Biol* **12**: 1959–1964.

Reboul J, Vaglio P, Tzellas N, *et al.* 2001. Open-reading-frame sequence tags (OSTs) support the existence of at least 17 300 genes in *C. elegans*. *Nature Genet* **27**: 332–336.

Roy PJ, Stuart JM, Lund J, Kim SK. 2002. Chromosomal clustering of muscle-expressed genes in *Caenorhabditis elegans*. *Nature* **418**: 975–979.

Stein L, Sternberg P, Durbin R, Thierry-Mieg J, Spieth J. 2001. WormBase: network access to the genome and biology of *Caenorhabditis elegans*. *Nucleic Acids Res* **29**: 82–86.

Tabara H, Grishok A, Mello CC. 1998. RNAi in *C. elegans*: soaking in the genome sequence. *Science* **282**: 430–431.

The *C. elegans* Sequencing Consortium 1998. Genome sequence of the nematode *C. elegans*: a platform for investigating biology. *Science* **282**: 2012–2018.

Timmons L, Court DL, Fire A. 2001. Ingestion of bacterially expressed dsRNAs can produce specific and potent genetic interference in *Caenorhabditis elegans*. *Gene* **263**: 103–112.

Urwin PE, Lilley CJ, Atkinson HJ. 2002. Ingestion of double-stranded RNA by preparasitic juvenile cyst nematodes leads to RNA interference. *Mol Plant Microbe Interact* **15**: 747–752.

Vaglio P, Lamesch P, Reboul J, *et al.* 2003. WorfDB: the *Caenorhabditis elegans* ORFeome Database. *Nucleic Acids Res* **31**: 237–240.

Walhout AJ, Reboul J, Shtanko O, *et al.* 2002. Integrating interactome, phenome, and transcriptome mapping data for the *C. elegans* germline. *Curr Biol* **12**: 1952–1958.

Walhout AJ, Sordella R, Lu X, *et al.* 2000. Protein interaction mapping in *C. elegans* using proteins involved in vulval development. *Science* **287**: 116–122.

Webb CT, Shabalina SA, Ogurtsov AY, Kondrashov AS. 2002. Analysis of similarity within 142 pairs of orthologous intergenic regions of *Caenorhabditis elegans* and *Caenorhabditis briggsae*. *Nucleic Acids Res* **30**: 1233–1239.

Wodarz A. 2002. Establishing cell polarity in development. *Nature Cell Biol* **4**: E39–44.

Copyright © 2003 John Wiley & Sons, Ltd. 
